# Early weight gain trajectories and body composition in infancy in infants born very preterm

**DOI:** 10.1111/ijpo.12752

**Published:** 2020-11-17

**Authors:** Victoria A.A. Beunders, Jorine A. Roelants, Jessie M. Hulst, Dimitris Rizopoulos, Anita C.S. Hokken‐Koelega, Esther G. Neelis, Kirsten S. de Fluiter, Vincent W.V. Jaddoe, Irwin K.M. Reiss, Koen F.M. Joosten, Marijn J. Vermeulen

**Affiliations:** ^1^ Department of Pediatrics, Division of Neonatology Erasmus MC–Sophia's Children's Hospital Rotterdam The Netherlands; ^2^ Department of Paediatrics, Division of Gastroenterology, Hepatology and Nutrition Hospital for Sick Children Toronto Canada; ^3^ Department of Biostatistics Erasmus MC Rotterdam The Netherlands; ^4^ Department of Pediatrics, Division of Pediatric Endocrinology Erasmus MC–Sophia's Children's Hospital Rotterdam The Netherlands; ^5^ Department of Pediatrics, Division of Pediatric Gastroenterology Erasmus MC–Sophia's Children's Hospital Rotterdam The Netherlands; ^6^ Department of Pediatrics Erasmus MC–Sophia's Children's Hospital Rotterdam The Netherlands; ^7^ Department of Pediatrics, Intensive Care Unit Erasmus MC–Sophia's Children's Hospital Rotterdam The Netherlands

**Keywords:** air‐displacement plethysmography, fat mass, lean mass, PEAPOD

## Abstract

**Background:**

Concerns are raised about the influence of rapid growth on excessive fat mass (FM) gain in early life and later cardiometabolic health of infants born preterm.

**Objectives:**

To study the association between postnatal weight gain trajectories and body composition in infancy in infants born very preterm.

**Methods:**

In infants born <30 weeks gestation, we evaluated associations between weight Z‐score trajectories for three consecutive timeframes (NICU stay, level‐II hospital stay and at home) and body composition, measured at 2 and 6 months corrected age by air‐displacement plethysmography.

**Results:**

Of 120 infants included, median gestational age at birth was 27^+5^ (interquartile range 26^+1^;28^+5^) and birth weight 1015 g (801;1250). The majority of infants did not make up for their initial loss of weight Z‐score, but growth and later body composition were within term reference values. Weight gain during NICU stay was not associated with fat mass (absolute, %FM or FM index) in infancy. Weight gain during NICU and level II hospital stay was weakly associated with higher absolute lean mass (LM), but not after adjustment for length (LM index). Weight gain in the level‐II hospital was positively associated with fat mass parameters at 2 months but not at 6 months. Strongest associations were found between weight gain at home and body composition (at both time points), especially fat mass.

**Conclusions:**

Weight gain in different timeframes after preterm birth is associated with distinct parameters of body composition in infancy, with weight gain at home being most strongly related to fat mass.

## 1 INTRODUCTION

Infants who are born prematurely start their extra‐uterine life in a critical period for growth and development.^1^, ^2^ They are at high risk for postnatal growth restriction, associated with long‐term neurodevelopmental problems.^3^, ^4^ To improve neurodevelopmental outcome, pro‐active nutritional treatment in early life has been recommended by the American Academy of Pediatrics and European Society of Paediatric Gastroenterology, Hepatology and Nutrition (ESPGHAN).^5^, ^6^ However, concerns have been raised about the adverse influence of high nutritional intake and rapid growth during the first months of life on later cardiometabolic health.^7^, ^8^ The underlying theory is covered in the Developmental Origins of Health and Disease (DOHaD) paradigm. The DOHaD hypothesis postulates that after a period of nutritional deprivation, stress, or inflammation (eg, fetal growth restriction (FGR), preterm birth or stay on the neonatal intensive care unit (NICU)), an environment with a relative excess of oxygen (radicals) and nutrients can result in an increased risk of adverse cardiometabolic health.^9^

In infants born at term, higher protein intake during the first year of life was found to be associated with an increased risk of obesity at school age.^10^ In infants born preterm, however, recent studies show contradictive effects of enhanced early nutrition and growth during the first year of life on long‐term cardiometabolic outcome.^11^, ^12^, ^13^, ^14^, ^15^, ^16^, ^17^ The question therefore remains which growth pattern is most beneficial for long‐term outcome: do the known neurodevelopmental benefits of early rapid weight gain outweigh the potential risk of adverse cardiometabolic health in child‐ and adulthood?^18^

To answer these questions, it is essential to determine whether early rapid weight gain in infants born preterm is indeed harmful, and to identify critical periods. This is important as specifically in preterm born infants, nutritional practices change heavily during the first months of life, and even short periods of altered growth may have great impact on later health and development.^14^ Also, a reliable early marker of cardiometabolic health is needed. Body composition, which can be measured patient‐friendly in the outpatient setting, has been used in earlier studies showing different trajectories in infants born preterm and full term.^11^, ^19^

In term born infants, the first three months of life are identified as most critical for the development of cardiometabolic risk factors during infancy such as overall fat and visceral fat.^20^, ^21^ Unfortunately, in infants born preterm, studies assessing growth over multiple timeframes are scarce, so a critical growth period has not been identified yet.^14^ In The Netherlands, national policy is to transfer preterm born infants from the NICU (a level‐III or level‐IV hospital) to a level‐II hospital as soon as they are stable, usually between 30 and 32 weeks of gestation, to stay there until discharge home. These clear cut offs between an early “critical” neonatal phase and a more "stable" phase facilitate studying growth over different time frames.

In this study, we aimed to study the association between postnatal weight gain during three different timeframes (NICU‐stay, level‐II hospital stay and at home) and body composition at 2 and 6 months corrected age in infants born very preterm (< 30 weeks of gestation). We hypothesized that associations are timeframe specific, with greater postnatal weight gain being associated with both higher lean and fat mass in infancy.

## 2 METHODS

This study is part of an ongoing prospective observational cohort study (BOND Study), conducted at the level IV NICU and the outpatient clinic of the Erasmus MC—Sophia Children's Hospital, Rotterdam, the Netherlands. Infants born before 30 weeks of gestation, admitted within 48 hours after birth, were eligible for inclusion in the study. Exclusion criteria included congenital anomalies (including chromosomal defects) that may interfere with growth, severe brain injury (ie, intraventricular hemorrhage grade III/IV and post‐hemorrhagic ventricular dilatation requiring lumbar or ventricular reservoir punctures), congenital infections, and perinatal asphyxia (umbilical cord pH < 7.00 and APGAR score at 5 minutes < 5). Data were collected between September 2014 and January 2018. The study is registered in the Netherlands Trial Register (NTR6024) and approved by the local ethical review board. Written parental informed consent was obtained before enrollment in the study.

### 2.1 Local nutrition protocol

During NICU stay, all infants were fed according to the parenteral and enteral ESPGHAN guidelines.^6^, ^22^, ^23^ In short, parenteral glucose administration was started directly after birth, with a minimum of 4 and maximum of 12 mg/kg/min. Amino acid administration was also started directly after birth at 2.4 g/kg/d and gradually increased to a target dose of 3.5 to 4.0 g/kg/d. Lipids were started the day after birth at 2.4 g/kg/d and gradually increased to a target dose of 2.5 to 3 g/kg/d. Enteral bolus feeding was started on the day of birth and increased daily.^24^ With this increasing daily enteral intake, parenteral nutrition was stepwise decreased and ceased at an enteral intake of 130 mL/kg/d.

Expressed breast milk was the first choice of enteral feeding. If not (sufficiently) available, preterm formula was supplemented (Nenatal start, Nutricia Advanced Medical Nutrition, Zoetermeer, the Netherlands), as donor milk was not available. Breast milk fortification was started at an enteral intake of 100 mL/kg/d (Breast Milk Fortifier, Nutricia Advanced Medical Nutrition, Zoetermeer, the Netherlands).

After transfer from the NICU, feeding regimens, including fortification and post‐discharge feeding, were determined according to local hospital guidelines. Dependent on the developmental stage and health status of the child, scheduled nasogastric tube feeding was gradually decreased, to reach breastfeeding or bottle feeding on demand at home. After discharge home, post‐discharge formula or prolongation of fortification was not given routinely. The treating neonatologist set the indication for fortification and post‐discharge feeding, based on individual growth trajectories, taking feeding mode, tolerance, and parental preferences into account.

### 2.2 Clinical data

Maternal characteristics and obstetrical and neonatal data were prospectively collected. In this study unlabeled use of age refers to age corrected for prematurity. Postnatal age was defined as days after birth, with the day of birth corresponding with day 1. At each study visit, parents filled out a questionnaire on their infants' feeding practices to collect data on type of feeding, fortification, and complementary feeding. Estimation of socio‐economic status was based on home address, using Z‐scores that summarize the local average income, low‐income rate, low educational level rate, and unemployment rate in the ZIP code area.^25^

### 2.3 Growth

Body weight, head circumference, and length measurements were performed as part of standard care according to local protocols. Length was not routinely measured during NICU and level‐II hospital stay, but was part of follow‐up anthropometry. Gestational age (GA) and sex‐corrected Z‐scores for weight were calculated at the following time points: birth, postnatal weight nadir (day with lowest postnatal weight), 30 weeks GA, transfer from NICU to level‐II hospital, discharge home, and at both outpatient clinic visits (2 and 6 months). Z‐scores were based on the Fenton growth charts from birth until discharge or 50 weeks GA, and on the World Health Organization (WHO) growth charts thereafter.^26^, ^27^, ^28^

### 2.4 Outpatient clinic visits

All patients attended the standard national neonatal follow‐up program for medical and neurodevelopmental assessment. Visits were planned around 2 months and 6 months corrected age. At both visits, body composition was measured for research purposes using air‐displacement plethysmography (PEA POD, Infant Body Composition System, COSMED). This validated method estimates fat mass as percentage of total body weight (%FM), absolute fat mass (FM) and lean mass (LM) by direct measurements of body volume and mass.^29^, ^30^ To correct body composition variables for small body size (such as expected in our study group), FM index (FMI; FM (kg) / length (m)^2^) and LM index (LMI; lean mass (kg) / length (m)^2^) were calculated by dividing the FM and LM by squared length.^31^ Age and sex corrected Z‐scores for body composition parameters were calculated based on average values from a large group of term born infants measured at our research center within the same time period.^32^ The body composition data contained no extreme outliers to exclude from analysis.

## 3 STATISTICAL ANALYSES

The first step was to model individual weight gain trajectories based on weight Z‐score within different time frames using linear mixed models. Because infants experience physiologic weight loss in the first days of life, the first weight gain trajectory was estimated from the moment of maximum postnatal weight loss (weight nadir, median day 5).^33^ Weight gain trajectories were studied within the following timeframes: (a) NICU stay: from initial postnatal weight nadir until transfer from NICU to level‐II hospital; (b) level‐II hospital stay: from admission to level‐II hospital until discharge home; (c) home stay: from discharge home until body composition measurement at 2 or 6 months at the outpatient clinic. The weight gain trajectories were modelled by using weight Z‐score (dependent variable) as the response over time with postnatal age at weight measurement as covariate (independent). Fit of the level‐II hospital and home weight gain statistical model was optimal with a random intercept and slope, and for the NICU weight gain model with addition of a quadratic slope. Subject‐specific weight Z‐score trajectories were then expressed by the individual intercept and (quadratic) slope. In the second step of the analysis, these subject‐specific weight Z‐score indicators were used as covariates (independent) in the linear regression analyses with %FM, FM, FMI, LM and LMI as outcome measures (dependent). The basic regression model included the subject‐specific weight Z‐score indicators, and sex, gestational age at birth, birth weight Z‐score, and corrected age at body composition measurement. The adjusted model included the covariates of the basic model plus days on parenteral nutrition during NICU stay, days on mechanical ventilation, socio‐economic status, and the use of any breast milk at the 2 months‐visit.

To evaluate the contribution of the weight Z‐score trajectories to body composition, we compared the explained variances of the model with and without the subject specific weight Z‐score indicators included, using the Likelihood Ratio Test. The difference in explained variance of the models could be contributed to the subject‐specific effects of weight gain.

### 3.1 Explorative analysis

To date, no strict values for body composition parameters are known to be considered harmful for long‐term health. Therefore, we divided our study population in three tertiles for FMI at 6 months. We visually explored the weight Z‐scores trajectories of these tertiles between birth and 6 months.

A 2‐tailed *P* value <.05 was considered statistically significant. Analyses were performed using SPSS package 25.0 (IBM SPSS Statistics, Armonk, NY) and R (R: A language and environment for Statistical Computing, version 3.May 1, 2018 for Windows, R Core Team, Vienna, Austria).

## 4 RESULTS

**FIGURE 1:**
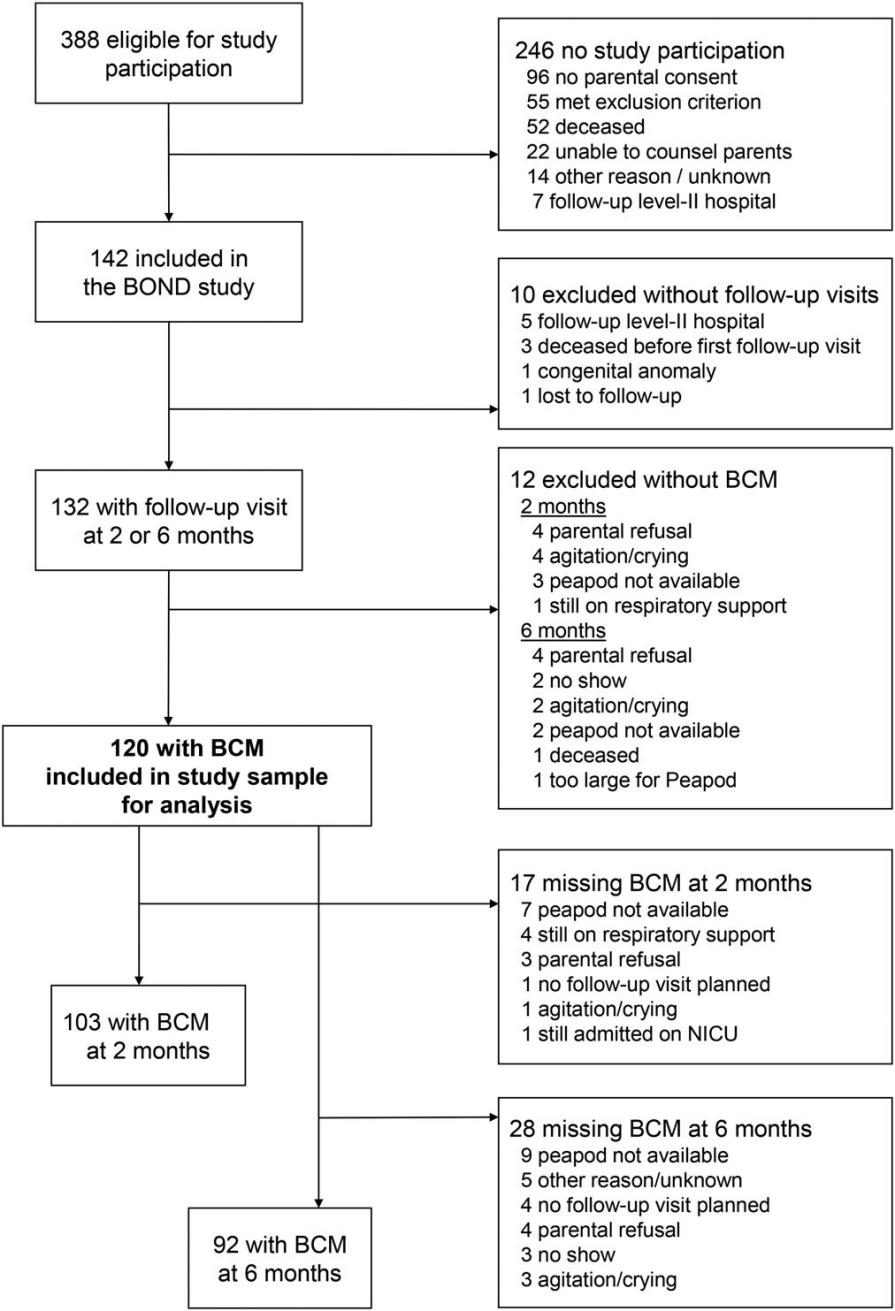
Flowchart of the study population. Flowchart of the study population and number of body composition measurements at the outpatient clinic visits at 2 and 6 months. Abbreviations: BCM, body composition measurement; NICU, neonatal intensive care unit

In total, 142 infants were enrolled in the BOND Study. After excluding one infant with a congenital anomaly interfering with growth (panhypopituitarism), three infants who deceased before discharge home, six without follow‐up visits at the outpatient clinic, and 12 without any body composition measurements, the study population included for analysis consisted of 120 infants **(**Figure 1 **)**.

### 4.1 Baseline characteristics

The baseline characteristics of the study population are provided in Table 1. Median GA at birth was 27^+5^ weeks (interquartile range [IQR] 26^+1^;28^+5^) with a birth weight of 1015 g (IQR 801;1250), 0.1 SD (−0.4;0.7). Median duration of parenteral amino acid or lipid administration during NICU stay was 10 days (8;16). The majority of the infants (*n* = 90, 75%) received both own mothers milk and formula during NICU stay. The infants were transferred from the NICU to a level‐II hospital at a median postnatal age of 29 (17;67) days, corresponding with 32^+0^ (30^+3^;36^+1^) weeks of gestation. They were discharged home at a postnatal age of 84 (70;104) days, corresponding with 39^+5^ (38^+0^;41^+5^) weeks of gestation.

**TABLE 1:** Maternal and infant characteristics (n = 120)

Maternal characteristics		
Age at delivery	*years*	30 (27;34)
Pre‐pregnancy BMI	*kg/m* ^*2*^	24.7 (21.8;29.1)^1^
Pregnancy complications	*(G)DM*	7 (6%)
	*Hypertension* ^a^	8 (7%)
	*PE/HELLP*	20 (17%)
	*FGR* ^b^	22 (18%)^2^
	*PPROM*	25 (21%)
Singleton pregnancy		94 (78%)
Antenatal corticosteroids	*0/1/2 doses*	8/33/79 (7/28/66%)
Caesarean section		70 (58%)
*Infant characteristics*		
Sex	*male*	75 (63%)
GA at birth	*weeks* ^*+days*^	27^+5^ (26^+1^;28^+5^)
Birth weight	*gram*	1015 (801;1250)
	*Z‐score*	0.1 (−0.4;0.7)
Apgar	*5 min*	8 (6;9)^3^
Culture‐proven sepsis	*early onset* ^c^	4 (3%)
	*late onset*	39 (33%)
NEC	*Bell stage ≥ 2*	5 (4%)
Treated PDA		39 (33%)
BPD ^f^	*mild*	28 (23%)
	*severe*	17 (14%)
Postnatal steroid use		20 (17%)^2^
Brain injury ^e^		36 (30%)
Treated ROP		6 (5%)
Mechanical ventilation	*days*	2 (0;11)
NICU stay	*days*	29 (17;67)
GA at NICU transfer to level‐II hospital	*weeks* ^*+days*^	32^+0^ (30^+3^;36^+1^)
Total hospital stay ^f^	*days*	84 (68;104)^3^
GA at discharge home	*weeks* ^*+days*^	39^+5^ (38^+0^;41^+5^)^3^

*Note:* All data are expressed in median (interquartile range) or number (percentages).

Abbreviations: BPD, bronchopulmonary dysplasia; BMI, body mass index; HELLP, hemolysis, elevated liver enzymes, and a low platelet count; FGR, fetal growth restriction; PPROM, preterm prelabour rupture of membranes; GA, gestational age; (G)DM, (gestational) diabetes mellitus; PE, pre‐eclampsia, n, number; NEC, necrotizing enterocolitis; NICU, neonatal intensive care unit; PDA, patent ductus arteriosus; ROP, retinopathy of prematurity.

^a^ Either pre‐existent or pregnancy induced.

^b^ Estimated fetal weight or abdominal circumference below 10th percentile on Robinson curve.

^c^ Positive blood culture within 72 hours after birth. BPD: >28 days O2 + X‐ray abnormalities, severe BPD: endotracheal or CPAP at 36 weeks of gestation or > 30% fiO2 or > 1 L/min flow via nasal prongs.

^e^ Brain injury includes IVH gr I/II, cerebellar bleeding, arterial/venous stroke, periventricular leukomalacia and convulsions.

^f^ NICU + level‐II hospital. Missing data: ^1^ 14 infants, ^2^ 4 infants, ^3^ 1 infant.

### 4.2 Growth

Growth data are shown in Table 2. Maximum initial weight loss of birth weight was median 10.4% (IQR 7.6;13.6) which was reached on median day five (3;6), resulting in a weight Z‐score decrease of −0.8 SD. Overall, infants were unable to make up for this early loss of weight Z‐score during the study period. Median length and head circumference did show an increase in Z‐score from −1.6 SD and − 0.8 SD at transfer to the level‐II hospital to −0.2 SD and + 0.3 SD at 6 months, respectively.

**TABLE 2:** Growth and body composition parameters from birth to 6 months

		Birth	Weight nadir	Transfer NICU to level‐II hospital	Discharge home	2 M‐visit	6 M‐visit
*Growth* ^*1*^	n	120	120	120	119	119	113
Gestational/corrected age^2^	*weeks* ^*+days*^	27^+5^ (26^+1^;28^+5^)	28^+2^ (26^+5^;29^+1^)	32^+0^ (30^+3^;36^+1^)	39^+5^ (38^+0^;41^+5^)^a^	7^+4^ (6^+4^;9^+6^)	26^+3^ (25^+2^;27^+5^)
Postnatal age	*days*	1	5 (3;6)	29 (17;67)	84 (68;104)^a^	138 (128;163)	273 (261;286)
Weight	*kg*	1.02 (0.80;1.25)	0.89 (0.73;1.10)	1.45 (1.25;2.04)	3.11 (2.71;3.59)^a^	4.73 (4.15;5.30)	7.19 (6.42;7.73)
	*Z‐score*	0.1 (−0.4;0.7)	−0.7 (−1.2;‐0.3)	−0.9 (−1.5;‐0.4)	−0.7 (−1.7;0.1)^a^	−0.8 (−1.6;‐0.1)	−0.7 (−1.5;0.1)
Head circumference	*cm*	24.8 (23.7;26.6)^b^	NA	28.0 (26.9;31.4)^c^	34.5 (33.2;36.3)^d^	38.7 (37.5;40.0)^b^	43.4 (42.0;44.2)^b^
	*Z‐score*	0.1 (−0.5;0.6)^b^	NA	−0.8 (−1.4;‐0.3)^c^	−0.1 (−1.1;0.6)^d^	0.2 (−0.7;1.0)^b^	0.3 (−0.5;1.0)^b^
Length	*cm*	NA	NA	38.0 (36.6;42.4)^e^	48.0 (46.0;50.3)^f^	55.2 (53.6;57.2)^a^	66.4 (64.0;68.5)^g^
	*Z‐score*	NA	NA	−1.6 (−2.6;−1.0)^e^	‐1.0 (−2.1;‐0.6)^f^	−1.1 (−1.9;‐0.3)^a^	−0.2 (−1.2;0.5)^g^
*Body composition*	**n**					103	92
Relative fat mass	*%*	NA	NA	NA	NA	21.9 (17.8;23.9)	20.4 (18.0;23.3)
	*Z‐score*	NA	NA	NA	NA	0.5 (−0.4;1.1)	−0.6 (−1.3;‐0.1)
Absolute fat mass	*kg*	NA	NA	NA	NA	0.98 (0.78;1.30)	1.44 (1.12;1.70)
	*Z‐score*	NA	NA	NA	NA	0.0 (−0.8; 0.8)	−0.8 (−1.5;‐0.2)
Fat mass index	*kg/m* ^*2*^	NA	NA	NA	NA	3.42 (2.66;3.95)	3.25 (2.76;3.87)
	*Z‐score*	NA	NA	NA	NA	0.3 (−0.4;1.1)	−0.6 (−1.2;‐0.1)
Absolute lean mass	*kg*	NA	NA	NA	NA	3.70 (3.33;4.04)	5.65 (5.08;6.08)
	*Z‐score*	NA	NA	NA	NA	−0.9 (−1.6;‐0.3)	−0.4 (−1.2;0.3)
Lean mass index	*kg/m* ^*2*^	NA	NA	NA	NA	12.04 (11.32;12.82)	12.67 (12.0;13.4)
	*Z‐score*	NA	NA	NA	NA	−0.1 (−0.8;0.8)	0.1 (−0.6;0.6)

*Note:* All data are expressed in median (interquartile range) or number (percentages). Length measurement was not routinely collected during NICU and level‐II hospital stay, leading to many missing values. Z‐scores for body composition parameters were calculated based on average values from a large group of term born infants assessed at our research center within the same time period. ^1^ Missing growth data of one infant at discharge home, one infant at 2 M‐visit (no appointment at outpatient clinic) and seven infants at 6 M‐visit (4 no appointment at outpatient clinic, 3 no show for appointment). ^2^ Gestational age for birth until discharge home, corrected age for 2 M‐ and 6 M‐visits.

Abbreviations: cm, centimeter; m, meter; M, month; NICU, neonatal intensive care unit; n, number; kg, kilograms.

Number of infants with missing data:

^a^ 2 Infants,

^b^ 3 Infants,

^c^ 14 Infants,

^d^ 44 infants,

^e^ 64 infants,

^f^ 71 infants,

^g^ 1 infant.

### 4.3 Body composition

Of the 120 infants enrolled in the study, body composition was measured in 103 (86%) at 2 months and in 92 (77%) at 6 months (Table 2 **)**. In 76 infants (63%), body composition was measured at both time points. Reasons for missing body composition measurements are presented in Figure 1.

Median %FM at 2 months was 21.9% (0.5 SD, 17.8;23.9) and 20.4% (−0.6SD, 18.0;23.3) at 6 months, with similar values in boys and girls (Table S1). Between 2 and 6 months, median FMI of all infants measured decreased whereas median LMI increased. Clinical and nutritional characteristics of the infants during the outpatient visits can be read from Table 3.

**TABLE 3:** Clinical characteristics at the outpatient clinic visits

		2 months	6 months
n		119 (99%)	113 (94%)
Corrected age	*weeks*	7.6 (6.6;9.9)	26.4 (25.3;27.7)
Feeding type^a^	*Own mothers milk*	19 (16%)	5 (4%)
	*Formula feeding*	78 (66%)	101 (89%)
	*Mixed feeding*	22 (19%)	4 (4%)
Enriched nutrition^b^		23 (20%)	4 (4%)
Tube feeding		16 (13%)	4 (4%)
Parenteral nutrition		2 (2%)	1 (1%)
Oxygen supply		9 (8%)	4 (4%)

*Note:* All data are expressed in median (interquartile range) or number (percentages). protein, or with fat or carbohydrates (rare); data missing for 1 infant at 2 months and 2 infants at 6 months.

Abbreviation: n; number.

^a^ Data missing for 3 infants at 6 months,

^b^ Either preterm formula or fortified human milk enriched with extra protein, or with fat or carbohydrates (rare), data missing for 1 infant at two months CA and 2 infants at six months CA.

### 4.4 Associations between weight gain and body composition

The results of the fully adjusted regression analyses are presented in Table 4. The basic model generally showed the same effects and similar effect sizes as the fully adjusted model (data not shown). NICU weight gain was weakly associated with higher LM at 2 months: weight gain (Z‐score) trajectories during NICU stay explained 3.3% of the variance in LM at 2 months, with the association losing significance when LM was adjusted for length (LMI). No association was found for NICU weight gain and FM, %FM or FMI at 2 months, or with any of the body composition parameters at 6 months.

**TABLE 4:** Associations between weight gain trajectories and body composition at 2 months and 6 months

	2 months (n = 103)					6 months (n = 92)				
	%FM	FM	FMI	LM	LMI	%FM	FM	FMI	LM	LMI
*Weight gain trajectory*										
NICU	¥	¥	¥	3.3%*	¥	¥	¥	¥	¥	¥
Level‐II hospital	6.5%*	11.9%**	7.9%*	10.8%**	¥	¥	¥	¥	4.6%*	¥
Home	32.6%**	49.8%**	46.3%**	24.6%**	6.0%*	24.6%**	46.8%**	36.5%**	31.8%**	8.6%*

*Note:* The values represent the variance (R^2^) in body composition explained by the weight gain trajectories (Z‐score), computed as the change in R^2^ by adding the timeframe specific weight gain trajectory indicators (intercept and/or slope) to the linear regression model. Covariates included gestational age at birth, birth weight Z‐score, sex, corrected age at body composition measurement, days on parenteral nutrition during NICU stay, days on invasive respiratory support, socio‐economic status, and breast milk at two months CA (any/no). ¥ R^2^ < 3% and *P*‐value >.05, * *p*‐value <.05, **p‐value <.001.

Abbreviations: %FM, percentage fat mass relative to weight; FM, absolute fat mass in kilograms; FMI, fat mass index (FM/length(m)^2^); LM, absolute lean mass in kilograms; LMI, lean mass index (LM/length(m)^2^); NICU, neonatal intensive care unit.

Weight gain in the level‐II hospital was positively associated with all components of body composition at 2 months, except LMI. At 6 months, it was only positively associated with absolute LM, not with LMI or any of the fat parameters. Weight gain at home was strongly positively associated with all measures of body composition at both 2 and 6 months, especially fat parameters. For FMI, the variance explained by weight gain at home was 46.3% at 2 months, and 36.5% at 6 months (both *P* < 0.001).

### 4.5 Explorative analyses

**FIGURE 2:**
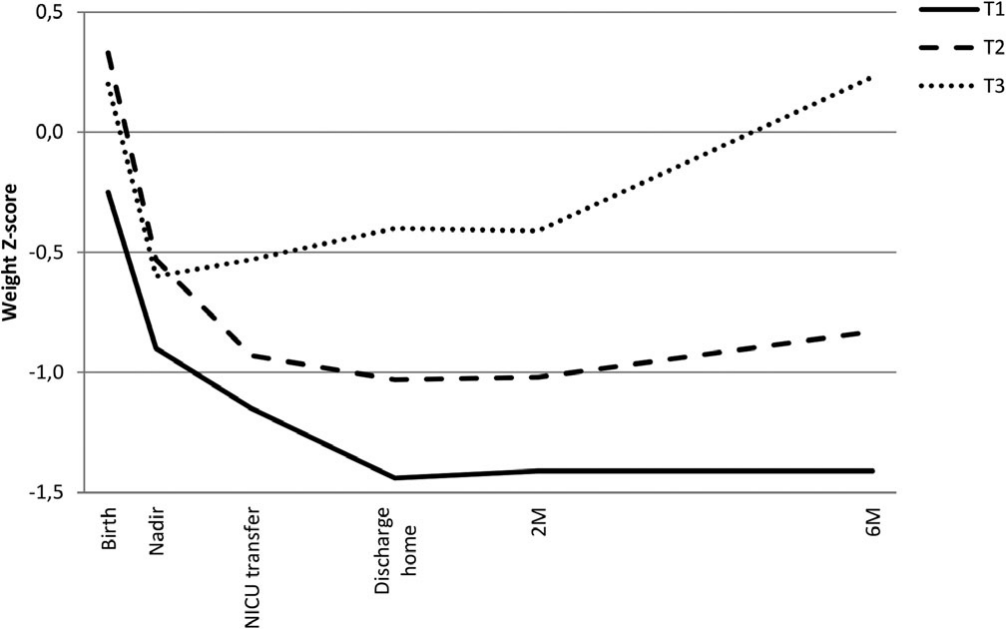
Weight Z‐score trajectories grouped for each FMI tertile at 6 months. Abbreviations: FMI, fat mass index; T1, lowest FMI tertile (FMI ≤2.93 kg/m^2^); T2, middle FMI tertile (FMI 2.93‐3.71 kg/m^2^); T3, highest FMI tertile (FMI ≥3.72 kg/m^2^); Nadir, day with lowest postnatal weight; NICU, neonatal intensive care unit; M, months

Median weight Z‐score trajectories from birth to 6 months of the three FMI tertiles at 6 months are shown in Figure 2, with infant characteristics and growth parameters of each subgroup in Table S2. In all groups, around 50% was being exclusively breastfed and 55% used fortification at discharge home. Infants in the lowest tertile started at a 0.5 SD lower mean birth weight Z‐score as compared to the middle and highest tertile, and overall lost another 1 SD during hospital stay, to remain stable after discharge home until 6 months. Their relatively lower weight at 6 months (−1.4 SD) is mainly explained by low fat (FMI ‐1.6 SD), at a normal lean mass for length (LMI 0.0 SD). Infants in the middle FMI tertile started at a higher birth weight Z‐score than infants in the lowest tertile, but showed a similar pattern with an initial drop to −1 SD in weight Z‐score and little change after NICU transfer. Their body composition measures suggest a more balanced fat and lean mass acquisition, with a FMI at −0.7 SD and LMI at −0.1 SD. Only the highest tertile showed a gradual increase of weight Z‐score after the initial dip in the NICU, with the steepest increase in the period between 2 and 6 months. Their growth and body composition measures were very close to the median of the healthy term born references, suggesting proportionate growth.

## 5 DISCUSSION

In this prospective cohort study we studied the associations between postnatal weight gain trajectories during different timeframes, and body composition in infancy in infants born very preterm. We found that weight gain in the postnatal period was associated with an increase in both lean and fat mass in infancy. In line with the hypothesis, the associations with body composition were timeframe specific. Greater weight gain during NICU and level II hospital stay was (weakly) associated with higher absolute lean mass in infancy, but not after correction for length (LMI). Weight gain during NICU stay was not associated with any of the fat mass parameters (absolute, %FM or FMI), though weight gain in the level‐II hospital was positively associated with all three fat mass parameters at 2 months. Weight gain at home was most strongly associated with body composition, especially fat mass, at both 2 and 6 months, also when adjusted for length.

Our findings are largely in line with previous studies, reporting positive associations between in‐hospital weight gain and lean mass and %FM at discharge.^34^, ^35^ With regard to body composition later in infancy, previous studies showed that early weight gain (from birth until body composition measurement) was associated with %FM at 3, 6, and 12 months,^11^ whereas insufficient weight gain before and after 36 weeks GA was associated with lower lean mass, fat mass and %FM at 6 months.^36^ What our study adds is more insight into effects of weight gain over different (critical and stable) timeframes in infancy on body composition. In addition, by using FMI and LMI, we took length into account, which is important in a cohort of preterm born children at risk of restricted growth.

Our longitudinal data showed that the postnatal decrease in median weight Z‐score from 0.1 SD to −0.7 SD in the first days of life, in clinical practice considered as physiological, was not recovered at 6 months. Head circumference and length increased in Z‐score after NICU discharge, reaching values similar to healthy term born infants at 6 months. The increase in length and head circumference Z‐scores corresponded with a similar increase in lean mass between 2 and 6 months, also reflected by a stable lean mass index.

We found that %FM and FMI were above average values of term born infants at 2 months (+0.5 SD and + 0.3 SD) but decreased to below average term values at 6 months (both −0.6 SD). This decreasing relative fat mass during infancy corresponds with previous findings showing that %FM is higher in infants born preterm around term equivalent age, and decreases to levels below those of infants born full term three to four months after term age.^11^, ^37^, ^38^, ^39^, ^40^, ^41^ It seems that the peak in fat mass presents earlier in infants born preterm (at around 3 months corrected age) than in infants born full term (at around 6 months).^32^, ^42^, ^43^ The mechanism of this altered fat trajectory in infants born preterm is not yet understood. Recent studies suggest that the rise in fat mass in the first months after birth is the result of adaptation to challenges of ex utero life, and may therefore be physiological in both infants born at term and preterm.^11^, ^34^, ^42^ Following this hypothesis, infants born preterm would simply start this transition “earlier” but at a comparable postnatal age as infants born at term.^11^ Two studies comparing infants born “late” and “early” preterm indeed showed that infants born "early" preterm had a higher %FM around 32 to 36 weeks GA and at term equivalent age than infants born “later” preterm.^44^, ^45^ In addition to early adaptation, differences in nutrition and early feeding practices between term infants (“natural” ad lib oral feeding) and preterm infants (“artificial” parenteral and tube feeding) will likely play a role in the altered fat trajectory and earlier fat peak seen in preterm infants. Body composition measurements closer to birth are needed to further explore the exact onset of rapid fat accumulation in infants born preterm. In practice, this is complicated as body composition measurements using the PEA POD can only be performed when the infant is weaned from respiratory support.

Our findings on the association between weight gain during NICU stay and body composition in infancy can only cautiously be extrapolated to clinical practice. Based on earlier reports, the observed lean mass gain without an effect on fat mass may be beneficial for both neurodevelopmental outcome and cardiometabolic health.^41^, ^46^, ^47^, ^48^ This supports recent adaptations in nutritional policy, including early high parenteral amino acid provision. However, the lack of an association between NICU weight gain and fat mass in the first 6 months could also reflect malnourishment during NICU stay. In the acute phase after birth, preterm born infants may need all caloric intake for vital energy expenditure, with little to none left for fat storage or catch up growth.^23^ Altogether, our data suggest that during the first critical period, focus should still be on further optimizing nutrition to prevent growth restriction and lean mass deficit, without major concerns about adverse effects on cardiometabolic health in the first months of life.^34^

Greater weight gain after NICU stay, especially at home, was strongly related to higher fat mass levels in infancy. For example, infants in the highest tertile of FMI at 6 months showed the greatest increase in weight Z‐score between 2 and 6 months, mostly explained by fat mass gain. Interestingly, this did not result in high fat mass parameters as compared to term born infants. Therefore, it is unclear whether this weight gain is an actual risk factor for long term cardiometabolic health.^11^ On one hand, the trajectory with rapid fat accumulation may program the body for an increased risk of metabolic complications in adult life. On the other hand, this rapid fat accumulation may be protective for later cardiometabolic health by compensating for the growth restriction which developed during the critical phase of NICU stay. It may even be the infants in the lowest FMI tertile that are most at risk, as their weight Z‐score trajectory is most deviant from infants born full term. Therefore, follow‐up of this cohort into school age and adulthood is warranted to provide a complete view on the influence of early postnatal growth on cardiometabolic health, and to link the growth trajectories with neurodevelopmental outcome. Definitions of optimal growth and body composition trajectories in infants born preterm are needed for a next step in clinical nutritional care: using outcome‐based targets for growth and body composition could be preferred over the current practice of targeting on reference values based on the distribution in the general population.

The strengths of our study include the prospective design and longitudinal growth measurements, which enabled us to model individual weight Z‐score trajectories, rather than only cross sectional growth measures. Furthermore, the national policy of transferring infants born preterm to a level‐II hospital at around 32 weeks GA facilitated studying growth over different timeframes (critical vs stable hospital phase vs at home) which turned out to have distinct effects on body composition. Lastly, comparison of body composition measures with healthy term born infants was possible by the availability of locally generated reference values. A few considerations should be taken into account when interpreting our study results. First, only in 63% of the infants body composition measurement was measured twice, which might hamper the comparability of the group analyses at 2 and 6 months. Second, we used the PEA POD to measure body composition, because it is a validated device that is feasible in clinical and research settings and is most patient‐friendly. ^29^, ^30^, ^49^ A drawback of this method however, is the lack of information on fat distribution (eg, subcutaneous vs visceral), while excessive visceral fat is considered an important risk factor of adverse cardiometabolic health.^50^ Third, this study was not designed to elucidate the underlying mechanisms and risk factors explaining the differences in early growth trajectories. The sample size did not allow for subgroup analysis to study, for example, the effects of sex, FGR or type of nutrition on the associations between weight gain and body composition. We were also not able to enter more potential confounders to the models, such as specific medication use (eg, steroids) or nutritional intake during each timeframe. However, by correcting for several perinatal, neonatal and sociodemographic factors in our models, we assume that the most important confounders have been covered. This is supported by the descriptive data, which do not suggest an important role of sex, breastfeeding or fortification in the association between weight gain and body composition. To further explore the role of these factors in future large cohort studies, requires more detailed longitudinal data and complex statistical models, preferably incorporating fetal growth, longitudinal type of feeding, total daily caloric intake (dependent on diet and appetite), physical activity, health status, and genetic factors. The only proper way to disentangle the complex causal relation between nutrition and growth, is to randomize between different strategies in intervention studies.

## 6 CONCLUSIONS

In this prospective cohort study of infants born very preterm, we found that weight gain in different timeframes after preterm birth was associated with distinct parameters of body composition in infancy. When adjusted for length, NICU weight gain was not associated with body composition parameters in the first months of life. In contrast, weight gain after NICU stay, especially at home, was associated with an increase in lean mass and, most strongly, fat mass. However, as fat mass parameters in infancy were still below average values of infants born full term, further research is needed to explore the association between early postnatal growth and cardiometabolic outcome later in life.

## CONFLICT OF INTERESTS

No conflict of interest was declared.

## AUTHORS CONTRIBUTIONS

MJV, JAR, IKMR, KFMJ, ACSHK, VWVJ, and JMH made substantial contributions to conception and design of the study. VAAB, JAR, and MJV collected, analyzed and interpreted data, and drafted the article. EGN and KSdF participated in data collection. DR was involved in data analysis. All authors revised the article critically for important intellectual content and had final approval of the submitted and published versions of the manuscript.

## Supporting information


**Table S1** Growth and body composition parameters per sex from birth to 6 months.
**Table S2** Infant characteristics per tertile of FMI at 6 months.
