# Supplementary material for: Early weight gain trajectories and body composition in infancy in infants born very preterm
Source: Pediatr Obes. 2020 Nov 17;16(6):e12752. doi: 10.1111/ijpo.12752 (PMC8244114; doi:10.1111/ijpo.12752)
Supplement: Supplementary file 1 — Table S1 Growth and body composition parameters per sex from birth to 6 months. Table S2 Infant characteristics per tertile of FMI at 6 months. [file IJPO-16-e12752-s001.pdf]

# Early weight gain trajectories and body composition in infancy in infants born very preterm.

**Victoria A.A. Beunders MD[1#], Jorine A. Roelants MD PhD[1#],** Jessie M. Hulst MD PhD[2], Dimitris Rizopoulos PhD[3], Anita C.S. Hokken-Koelega MD PhD[4], Esther G. Neelis MD PhD[5], Kirsten S de Fluiter MD[4], V.W.V. Jaddoe MD PhD[6], Irwin K.M. Reiss MD PhD[1], Koen F.M. Joosten MD PhD[7], Marijn J. Vermeulen MD PhD[1*]

**# *Authors contributed equally***

[1] *Department of Pediatrics, Division of Neonatology, Erasmus MC – Sophia's Children's Hospital, Rotterdam, the Netherlands.*

[2] *Department of Paediatrics, Division of Gastroenterology, Hepatology and Nutrition, Hospital for Sick Children, Toronto, Canada.*

[3] *Department of Biostatistics, Erasmus MC, Rotterdam, the Netherlands.*

[4] *Department of Pediatrics, Division of Pediatric Endocrinology, Erasmus MC – Sophia's Children's Hospital, Rotterdam, the Netherlands.*

[5] *Department of Pediatrics, Division of Pediatric Gastroenterology, Erasmus MC – Sophia's Children's Hospital, Rotterdam, the Netherlands.*

[6] *Department of Pediatrics, Erasmus MC – Sophia's Children's Hospital, Rotterdam, the Netherlands.*

[7] *Department of Pediatrics, Intensive Care Unit, Erasmus MC – Sophia's Children's Hospital, Rotterdam, the Netherlands.*

*Corresponding author:

Marijn J Vermeulen, MD PhD
Erasmus MC – Sophia Children's Hospital
Dept. of Pediatrics, Div. of Neonatology, room Sk-3216
PO Box 2060
3000 CB Rotterdam, the Netherlands
Tel: +31 10 703 6077 / Fax: +31 10 703 6811
E-mail: m.j.vermeulen@erasmusmc.nl

**Table S1 | Growth and body composition parameters per sex from birth to 6 months**

## Hospital stay

| | | GIRLS | | | | BOYS | | | |
|---|---|---|---|---|---|---|---|---|---|
| | | **Birth** | **Weight nadir** | **Transfer from NICU** | **Discharge home** | **Birth** | **Weight nadir** | **Transfer from NICU** | **Discharge home** |
| | **n** | **45** | **45** | **45** | **45** | **75** | **75** | **75** | **72** |
| **Weight** | *kg* | 0.90 (0.74;1.12) | 0.80 (0.69;1.02) | 1.30 (1.15;1.93) | 2.91 (2.55;3.41) | 1.09 (0.85;1.29) | 0.94 (0.77;1.13) | 1.54 (1.30;2.28) | 3.19 (2.89;3.66) |
| | *Z-score* | -0.3 (-0.9;0.4) | -0.9 (-1.4;-0.4) | -1.0 (-1.7;-0.5) | -1.2 (-2.0;-0.1) | 0.3 (-0.1;0.7) | -0.6 (-1.0;-0.2) | -0.7 (-1.4;-0.4) | -0.6 (-1.6;0.2) |
| **Head circumference** | *cm* | 24.0 (23.3;26.4) | NA | 27.6 (26.0;30.3)[a] | 34.4 (33.0;36.2)[b] | 25.7 (24.0;27.0)[c] | NA | 28.4 (27.0;32.0)[d] | 35.0 (33.9;36.3)[e] |
| | *Z-score* | -0.1 (-0.8;0.5) | NA | -0.9 (-1.7;-0.4)[a] | -0.5 (-1.6;0.4)[b] | 0.2 (-0.4;0.6)[c] | NA | -0.6 (-1.2;-0.3)[d] | -0.7 (-1.7;0.1)[e] |
| **Length** | *cm* | NA | NA | 38.0 (37.0;42.2)[e] | 48.0 (46.0;51.0)[f] | NA | NA | 39.0 (36.5;42.5)[g] | 48.0 (45.0;50.0)[h] |
| | *Z-score* | NA | NA | -1.3 (-2.8;-0.6)[e] | -1.0 (-2.5;-0.7)[f] | NA | NA | -1.7 (-2.3;-1.2)[g] | -1.0 (-2.0;-0.5)[h] |

## Out-patient clinic

| | | GIRLS | | BOYS | |
|---|---|---|---|---|---|
| | | **2 months CA** | **6 months CA** | **2 months CA** | **6 months CA** |
| | **n** | **45** | **44** | **74** | **69** |
| **Weight** | *kg* | 4.39 (3.86;4.86) | 6.74 (6.06;7.47) | 4.88 (4.35;5.52) | 7.38 (6.62;8.09) |
| | *Z-score* | -1.2 (-1.7;-0.2) | -0.7 (-1.6;0.1) | -0.7 (-1.5;0.0) | -0.7 (-1.4;0.1) |
| **Head circumference** | *cm* | 37.5 (36.5;39.0)[i] | 42.4 (41.0;43.6)[j] | 39.1 (38.2;40.3)[j] | 43.8 (42.7;44.8)[i] |
| | *Z-score* | -0.2 (-1.0;0.9)[i] | 0.1 (-0.9;1.0)[j] | 0.4 (-0.6;1.2)[j] | 0.4 (-0.5;1.2)[i] |
| **Length** | *cm* | 54.5 (52.0;56.4)[i] | 65.4 (62.3;67.2) | 56.1 (54.0;57.4) | 67.1 (64.7;68.9)[j] |
| | *Z-score* | -1.3 (-2.3;-0.3)[i] | -0.2 (-1.4;0.5) | -1.0 (-1.8;-0.2) | -0.4 (-1.0;0.5)[j] |
| | **n** | **38** | **41** | **65** | **51** |
| **Relative fat mass** | *%* | 21.9 (17.6;23.5) | 20.5 (18.2;23.7) | 22.0 (18.3;24.0) | 20.2 (17.9;23.2) |
| | *Z-score* | 0.4 (-0.6;1.0) | -0.8 (-1.3;-0.3) | 0.6 (-0.2;1.2) | -0.5 (-1.0;0.0) |
| **Absolute fat mass** | *kg* | 0.95 (0.75;1.13) | 1.43 (1.07;1.69) | 1.06 (0.78;1.35) | 1.45 (1.24;1.73) |
| | *Z-score* | -0.3 (-0.9; 0.7) | -0.8 (-1.6;-0.3) | 0.1 (-0.7; 0.9) | -0.7 (-1.2;-0.2) |
| **Fat mass index** | $kg/m^2$ | 3.15 (2.66;3.79) | 3.08 (2.69;3.97) | 3.46 (2.56;4.06) | 3.39 (2.76;3.77) |
| | *Z-score* | 0.2 (-0.5;1.1) | -1.0 (-1.4;-0.2) | 0.4 (-0.4;1.3) | -0.5 (-1.1;-0.0) |
| **Absolute lean mass** | *kg* | 3.46 (3.25;3.78) | 5.25 (4.80;5.96) | 3.82 (3.42;4.20) | 5.85 (5.40;6.31) |
| | *Z-score* | -0.8 (-1.5;-0.1) | -0.5 (-1.6;0.3) | -1.0 (-1.7;-0.3) | -0.4 (-0.8;0.2) |
| **Lean mass index** | $kg/m^2$ | 11.91 (11.14;12.61) | 12.27 (11.81;12.97) | 12.32 (11.56;13.10) | 12.67 (12.0;13.4) |
| | *Z-score* | 0.0 (-0.8;0.9) | 0.0 (-0.6;0.8) | -0.1 (-0.9;0.8) | 0.2 (-0.6;0.6) |

**Legend**: All data are expressed in median (interquartile range). Length measurement was not routinely collected during NICU and level-II hospital stay, leading to incomplete data. Z-scores for body composition parameters were calculated based on average values from a large group of term born infants assessed at our research center within the same time period. Number of infants with missing data: [a] 5, [b] 20, [c] 3, [d] 9, [e] 22, [f] 27, [g] 42, [h] 41, [i] 2, [j] 1.

**Abbreviations:** NICU, neonatal intensive care unit; CA, corrected age; n, number; kg, kilograms; cm, centimeter; m, meter.

**Table S2 | Infant characteristics per tertile of FMI at 6 months**

| | T1 (FMI <2.93 kg/m$^2$) (n=30) | T2 (FMI 2.93-3.71 kg/m$^2$) (n=31) | T3 (FMI ≥3.72 kg/m$^2$) (n=31) |
|---|---|---|---|
| Hospital stay | | | |
| Sex (female) | 16 (53%) | 10 (32%) | 15 (48%) |
| GA at birth (weeks) | 27.6 [26.2;28.3] | 27.7 [27.3;28.9] | 28.0 [26.1;29.0] |
| Birth weight (grams) | 880 [790;1093] | 1080[965;1250] | 1058 [750;1315] |
| Birth weight SD | -0.3 [-0.9;0.7] | 0.3 [-0.3;0.6] | 0.2 [-0.3;0.9] |
| Birth head circumference SD | | | |
| Culture-proven sepsis [a] | 10 (33%) | 11 (36%) | 12 (39%) |
| NEC | 2 (7%) | 2 (7%) | 2 (7%) |
| Treated PDA | 10 (33%) | 9 (29%) | 10 (32%) |
| BPD [b], of which | 15 (50%) | 12 (39%) | 7 (23%) |
| - Mild | 8 (30%) | 9 (24%) | 5 (19%) |
| - Severe | 7 (27%) | 3 (3%) | 2 (9%) |
| Postnatal steroid use | 7 (23%) | 6 (19%) | 4 (13%) |
| Brain injury [c] | 9 (30%) | 9 (29%) | 12 (39%) |
| Mechanical ventilation (days) | 2 [0;7] | 0 [2;11] | 0 [0;4] |
| Parental nutrition (days) | 11 [9;17] | 10 [8;14] | 9 [7;14] |
| Total hospital stay (days) [d] | 84 [68;104] | 85 [70;100] | 75 [61;100] |
| NICU transfer weight SD | -1.2 [-1.6;-0.5] | -0.9 [-1.6;-0.5] | -0.5 [-1.2;-0.2] |
| NICU transfer head circumference SD | -1.0 [-1.6;-0.6] | -0.7 [-1.0;-0.3] | -0.4 [-1.5;0.1] |
| NICU transfer length SD | -1.4 [-2.3;-0.5] | -1.6 [-3.1;-1.1] | -1.2 [-2.7;-0.2] |
| Discharge home | | | |
| Feeding type | | | |
| - Only MM | 13 (48%) | 14 (50%) | 13 (48%) |
| - Only Formula | 9 (33%) | 8 (29%) | 7 (26%) |
| - Mix MM/Formula | 5 (19%) | 6 (21%) | 7 (26%) |
| Fortification | 14 (56%) | 15 (54%) | 13 (54%) |
| Tube feeding | 17 (63%) | 10 (35%) | 10 (36%) |
| Oxygen | 6 (20%) | 2 (7%) | 1 (3%) |
| Weight SD | -1.4 [-1.8;-0.3] | -1.0 [-1.9;-0.1] | -0.4 [-1.5;0.3] |
| Head circumference SD | -0.7 [-1.8;0.7] | -0.2 [-1.1;0.8] | -0.1 [-1.1;0.6] |
| Length SD | -1.0 [-2.0;-0.6] | -0.9 [-3.0;-0.5] | -1.1 [-2.1;-0.3] |
| 2-month visit | | | |
| Feeding type | | | |
| - Only MM | 3 (10%) | 8 (26%) | 3 (10%) |
| - Only Formula | 20 (67%) | 18 (58%) | 21 (70%) |
| - Mix MM/Formula | 7 (23%) | 5 (16%) | 6 (20%) |
| Fortification | 8 (27%) | 5 (16%) | 5 (17%) |
| Tube feeding | 7 (23%) | 5 (16%) | 3 (10%) |
| Oxygen | 5 (17%) | 3 (10%) | 1 (3%) |
| Weight SD | -1.4 [-2.1;-0.7] | -1.0 [-1.8;0.0] | -0.4 [-1.1;0.4] |
| Head circumference SD | -0.3 [-1.2;1.0] | 0.6 [-0.6;1.0] | 0.2 [-0.8;1.3] |
| Length SD | -1.2 [-2.1;-0.2] | -1.3 [-2.1;-0.3] | -0.7 [-1.9;-0.3] |
| FMI SD | -0.4 [-1.1;0.3] | 0.4 [-0.4;1.1] | 0.8 [-0.1;1.8] |
| LMI SD | -0.7 [-1.3;0.4] | -0.1 [-0.9;0.8] | 0.4 [-0.5;1.1] |
| 6-month visit | | | |
| Feeding type | | | |
| - Only MM | 1 (3%) | 3 (10%) | 1 (3%) |
| - Only Formula | 29 (97%) | 27 (87%) | 26 (90%) |
| - Mix MM/Formula | 0 (0%) | 1 (3%) | 2 (7%) |
| Fortification | 1 (4%) | 2 (7%) | 1 (4%) |
| Tube feeding | 0 (0%) | 1 (3%) | 2 (7%) |
| Oxygen | 3 (10%) | 1 (3%) | 0 (0%) |
| Weight SD | -1.4 [-2.1;-0.8] | -0.8 [-1.6;-0.3] | 0.2 [-0.5;0.7] |
| Head circumference SD | -0.1 [-1.2;0.8] | 0.7 [-0.2;1.2] | 0.5 [-0.4;1.4] |
| Length SD | -0.5 [-1.3;0.3] | -0.5 [-1.0;0.5] | 0.0 [-1.2;0.7] |
| FMI SD | -1.6 [-2.0;-1.2] | -0.7 [-0.9;-0.4] | 0.2 [-0.1;0.7] |
| LMI SD | 0.0 [-0.7;0.5] | -0.1 [-1.0;0.6] | 0.3 [-0.4;1.4] |

**Legend**: All data are expressed in median (interquartile range) or number (percentages).
[a] Positive blood culture within 72h after birth; [b] BPD: >28 days O2 + X-ray abnormalities, severe BPD: endotracheal or CPAP at 36 weeks of gestation or >30% fiO2 or >1L/min flow via nasal prongs; [c] Brain injury includes IVH gr I/II, cerebellar bleeding, arterial/venous stroke, periventricular leukomalacia and/or convulsions; [d] NICU + level-II hospital.
**Abbreviations**: FMI, fat mass index (FM/height$^2$ in meters); n, number; GA, gestational age; NEC, necrotizing enterocolitis; PDA, patent ductus arteriosus; BPD, bronchopulmonary dysplasia; NICU, neonatal intensive care unit; MM, mothers milk.
